# Personal exposure to air pollution and respiratory health of COPD patients in London

**DOI:** 10.1183/13993003.03432-2020

**Published:** 2021-07-15

**Authors:** Dimitris Evangelopoulos, Lia Chatzidiakou, Heather Walton, Klea Katsouyanni, Frank J. Kelly, Jennifer K. Quint, Roderic L. Jones, Benjamin Barratt

**Affiliations:** 1 Environmental Research Group, MRC Centre for Environment and Health, Imperial College London, London, UK; 2 National Institute for Health Research Health Protection Research Unit in Environmental Exposures and Health, Imperial College London, London, UK; 3 Centre for Atmospheric Science, Dept of Chemistry, University of Cambridge, Cambridge, UK; 4 Dept of Hygiene, Epidemiology and Medical Statistics, Medical School, National and Kapodistrian University of Athens, Athens, Greece; 5 National Heart and Lung Institute, Imperial College London, London, UK

## Abstract

Previous studies have investigated the effects of air pollution on chronic obstructive pulmonary disease (COPD) patients using either fixed-site measurements or a limited number of personal measurements, usually for one pollutant and a short time period. These limitations may introduce bias and distort the epidemiological associations as they do not account for all the potential sources or the temporal variability of pollution.

We used detailed information on individuals’ exposure to various pollutants measured at fine spatiotemporal scale to obtain more reliable effect estimates. A panel of 115 patients was followed up for an average continuous period of 128 days carrying a personal monitor specifically designed for this project that measured temperature, nitrogen dioxide (NO_2_), ozone (O_3_), nitric oxide (NO), carbon monoxide (CO), and particulate matter with aerodynamic diameter <2.5 and <10 μm at 1-min time resolution. Each patient recorded daily information on respiratory symptoms and measured peak expiratory flow (PEF). A pulmonologist combined related data to define a binary variable denoting an “exacerbation”. The exposure–response associations were assessed with mixed effects models.

We found that gaseous pollutants were associated with a deterioration in patients’ health. We observed an increase of 16.4% (95% CI 8.6–24.6%), 9.4% (95% CI 5.4–13.6%) and 7.6% (95% CI 3.0–12.4%) in the odds of exacerbation for an interquartile range increase in NO_2_, NO and CO, respectively. Similar results were obtained for cough and sputum. O_3_ was found to have adverse associations with PEF and breathlessness. No association was observed between particulate matter and any outcome.

Our findings suggest that, when considering total personal exposure to air pollutants, mainly the gaseous pollutants affect COPD patients’ health.

## Abstract 2

**Significant adverse associations were found between the respiratory health of COPD patients and their personal exposure to gaseous pollutants measured using portable sensors over 6 months. No significant associations were found for particulate pollutants.** https://bit.ly/3aqMT6O

## Introduction

Chronic obstructive pulmonary disease (COPD) is the third leading cause of death worldwide, with prediction of further increases unless urgent action is taken to reduce the underlying risk factors [1, 2]. COPD patients are at risk of acute episodes of deterioration (“exacerbations”) typically defined as a sudden worsening of respiratory symptoms. COPD exacerbations are the second commonest cause of adult emergency medical hospital admission in the UK, and are associated with increased mortality and decreased quality of life [3].

COPD hospitalisations and mortality increase in periods of high air pollution, and air pollutants at levels even within current guidelines may increase exacerbation risk [4–6]. Pollutants may trigger airway inflammation, thus leading to an exacerbation, or may increase susceptibility to viral/bacterial infection, increasing the severity and frequency of episodes [7]. The impact of extremes of, or rapid changes in, air pollution are not well characterised due, in part, to insufficiently detailed individual environmental exposure estimates in studies [8].

Human exposure to environmental stress in urban environments depends on a range of influencing factors, such as activity, surroundings, geography, proximity to source and meteorology. An individual's level of exposure is constantly changing as they go about their daily life and move through the environment. Traditionally, short-term exposure assessments of epidemiological studies are based on fixed monitoring locations, which cannot be used to accurately describe human exposure that comprises both indoor- and outdoor-generated pollution without many assumptions, which may introduce bias [9]. Active mobile sensors have been used in “snapshot” studies of exposure in micro-environments such as transport modes or indoors [10]. They have also been used to measure exposure in panel studies [11]. However, in each case monitoring periods were limited to a small number of hours or days and the number of subjects was small. Advances in measurement, materials and computing technologies mean that practical limitations are becoming less restrictive, presenting new opportunities for personal exposure assessment [12]. This development creates the potential for associations between environmental exposure and acute health outcomes to be assessed within large pre-selected panels or cohorts in usual daily environments, rather than in chamber or small panel studies. Furthermore, these sensors can now be deployed over much longer periods of time, allowing the capture of associations within a patient's normal daily routine and across seasons when behaviours may change.

The COPE study (www.erg.ic.ac.uk/research/home/projects/COPE-Characterisation-of-COPD-Exacerbations-using-Environmental-Exposure-Modelling.html) aimed to characterise associations between personal air pollution exposure and lung function, COPD symptoms and exacerbations within a cohort of 115 COPD patients. For the first time, direct personal exposure measurements were utilised for multiple pollutants, rather than proxy measurements or models, through the deployment of multiparameter personal sensors to all patients for up to 6 months each. By extending the period of personal exposure monitoring, infrequent health signals and symptoms could be investigated under a broad range of conditions and activities.

## Methods

A full study protocol for exposure data collection is available in Moore *et al*. [13]. A description of the study recruitment methodology and outcomes is described in Quint *et al*. [14]. Key details are described here.

### Participant sample

In total, 130 ex-smoking COPD patients were recruited through the Clinical Practice Research Datalink (CPRD; www.cprd.com) within Greater London in the UK, of whom 115 provided exposure and health data; 15 patients who took part in the study for <2 weeks were excluded. Patients were selected for recruitment based on prior medical history and were not housebound. Upon recruitment, patients filled out a basic questionnaire including type of residence, cooking and heating fuel, car ownership, and presence of smokers in the household, and were provided with a personal air quality monitor (PAM). A research physiotherapist trained participants in the use of a peak flow meter (PFM) and diary card at recruitment, followed up with a monthly telephone call. This level of interaction minimised dropout rates, and maximised valid data capture and diary card use.

### Personal air quality monitor

The PAM was designed, manufactured and tested specifically for the study. It is an autonomous unit that incorporates multiple sensors for activity and for physical and chemical parameters. The time resolution of the measurements was set at 20 s. Gaseous pollutants (nitrogen dioxide (NO_2_), ozone (O_3_), nitric oxide (NO) and carbon monoxide (CO)) were quantified with electrochemical sensors. Masses of particulate matter with aerodynamic diameter <2.5 and <10 μm (PM_2.5_ and PM_10_, respectively) were derived from particle counts taken by an optical particle counter. The PAM also recorded temperature, humidity, noise levels, three-dimensional accelerometery and Global Positioning System position. Measurements were aggregated to 1-min means and stored in a relational database. A description of the operation and performance of the PAM is described in Chatzidiakou *et al*. [15].

All sensors were calibrated in the winter and summer season, before and after the deployment to participants in outdoor co-locations with reference instruments. The root mean square error was less than 4 ppb for NO, NO_2_ and O_3_, 0.03 ppm for CO, and 9 and 2 μg·m^−3^ in winter and summer, respectively, for particulate matter.

No interaction with the PAM unit was required by the participant, other than to place it in its charger each night. Patients were asked to wear the PAM continuously for 6 months whenever they left their home. When at home, the PAM was placed within their living room.

### Health outcomes

Each evening, the participants filled out a diary card indicating any worsening of symptoms, any change in medication, including the use of oral steroids or antibiotics, and disrupted sleep patterns [16]. The diary card also asked whether the patient had left their home at any point and whether they had taken the PAM with them. Additionally, patients recorded their daily peak expiratory flow (PEF) using the PFM at the same time each day.

Upon completion of the fieldwork, diary cards were verified by a respiratory clinician and daily “exacerbation” events noted. Exacerbation was defined as a sustained worsening of symptoms (breathless, cough or sputum volume/colour) for at least 2 days beyond normal variation [17]. A change in treatment or healthcare utilisation without recorded symptoms was also considered.

The confirmation of each participant exacerbation was determined initially *via* telephone conversation follow-up and then again during a clinic visit, based on what was written on the diary card. Exacerbations were then re-confirmed by independent review of diary data by A.L. and J.K.Q. followed by a discussion of discrepancies in clinical opinion. Index of Multiple Deprivation (IMD) rank was assigned to each patient based on their home post code cross-referenced against 2015 UK census data [18].

### Statistical analysis

Pollutant concentrations were aggregated to daily means (CO, NO, NO_2_, PM_2.5_ and PM_10_) or daily maximum 8-h mean (O_3_). We applied a predefined mixed effects models analysis [19]. PEF measurements were analysed using linear regression, while the occurrences of exacerbation and daily symptoms, coded as binary variables, were examined by logistic regression. Random intercept models were used to provide conditional estimates on the expected change in the health outcomes, taking into account the correlation between each participant's repeated measurements [20]. Our core models were of the following form:
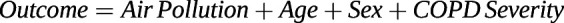
where *Air Pollution* refers to that individual's daily exposure to each of the pollutants in turn. To account for potential confounding effects, the full model included IMD as an indicator of the participants’ socioeconomic status and a binary variable for inhaled corticosteroid medication use. A restricted cubic spline of time with four degrees of freedom was introduced to account for potential long-term patterns in the data. Temperature was also included as a natural spline with three degrees of freedom.

To quantify the potential prolonged effects of air pollution on patients’ health, we assessed the effect estimates of lag 1, 2 and 3 days, and also the average lag 0–3 days. Two-pollutant models were also fitted with all pairwise combinations of pollutants except PM_2.5_–PM_10_, as the former is a subset of the latter, to account for the potential synergistic or confounding effects between pollutants. As sensitivity analyses, the full models were fitted excluding those person-days that patients left their home without taking their PAM with them. We investigated potential effect modification by season and COPD severity. For the former, we fit the full models in four categories, *i.e.* winter, spring, summer and autumn, while for the latter we included an interaction term coded into two categories, *i.e.* mild/moderate and severe/very severe. Finally, we carried out a comparison of the effect estimates yielded when ambient measurements from a central monitor were used as the exposure metrics.

The regression estimates were expressed as change in the expected PEF or as odds ratios for exacerbation and symptoms for a unit increase in all pollutants except CO, for which it was a 0.01 ppm increase. Estimates per interquartile range (IQR) are also reported. Missing data were excluded from the analysis. Stata version 15 (StataCorp, College Station, TX, USA) was used for all analyses.

### Ethics

The Research Ethics Committee for Camden and Islington provided ethical approval for the study (14/LO/2216). Approval was also granted by NHS Research and Development and the use of CPRD GOLD data was approved by the CPRD Independent Scientific Advisory Committee.

## Results

### Descriptive statistics

Mean age at recruitment was 70.5 years and the majority of patients (73%) suffered from moderate or severe COPD (table 1). All patients reported their primary address to be within Greater London.

**TABLE 1:** Summary statistics of peak expiratory flow (PEF), person-days recorded with symptoms according to patients’ diaries and baseline characteristics from the questionnaire

**Health outcomes (14 740 person-days)**	
PEF^#^ L·min^−1^	249±108
Exacerbation (yes)	2245 (15.2)
Breathlessness (yes)	2650 (18.0)
Cough (yes)	2285 (15.5)
Sleep disturbance (yes)	1528 (10.4)
Sputum (yes)	1227 (8.3)
Wheeze (yes)	1470 (10.0)
**Baseline characteristics (115 participants)**	
Female	54 (47.0)
Age years	70.5±8.1
COPD severity^¶^	
Mild	19 (16.5)
Moderate	52 (45.2)
Severe	32 (27.8)
Very severe	12 (10.5)
IMD rank^+^	16 528.2±8233.4
Medication use: ICS (yes)	80 (69.6)
Days each participant goes out per 2 weeks (0, 1, …, 14)	10.5±3.2
Car ownership (yes)	73 (63.5)
House type	
Flat	47 (40.9)
Semidetached/detached	37 (32.2)
Other	31 (27.0)
Cooking	
Gas	44 (38.3)
Electric	48 (41.7)
Gas hob/electric oven	20 (17.4)
Other including wood burning	3 (1.6)

Data are presented as mean±sd or n (%). COPD: chronic obstructive pulmonary disease; ICS: inhaled corticosteroid; IMD: Index of Multiple Deprivation. ^#^: maximum of three measurements per day; ^¶^: as defined by airflow obstruction; ^+^: overall 2015 IMD ranking in London from 586 to 32 700 and in this study from 1778 to 30 472.

Our database included 14 740 person-days from May 2015 to October 2017, with a mean and median follow-up of 128 and 145 days, respectively, per participant (minimum–maximum 14–208 days). An exacerbation was observed in 15% of the person-days, breathlessness in 18%, cough in 16%, sleep disturbance and wheeze in 10%, and sputum in 8%. The most common cause of data loss was participant withdrawal from the study. Other reasons included periods of participant vacation away from home, PAM malfunction, failure to charge the PAM and failure to record health data.

Mean±sd 24-h average personal exposure measurements were 13.8±6.3 ppb for NO_2_, 9.0±6.5 ppb for NO, 0.2±0.1 ppm for CO, 15.8±18.0 µg·m^−3^ for PM_2.5_ and 16.8±20.6 µg·m^−3^ for PM_10_, while the mean±sd 8-h maximum for O_3_ was 6.5±5.3 ppb (table 2). Seasonal differences were observed in pollutant concentrations (supplementary table S2). Temperature was relatively stable across the cohort, reflecting the fact that most participants spent most of their time inside temperature-controlled buildings.

**TABLE 2:** Descriptive statistics for personal measurements of air pollution and temperature

**Exposure**	**Valid observations n**	**Mean±sd**	**25th percentile**	**Median**	**75th percentile**	**Between-patient sd**	**Within-patient sd**	**Capture rate %^#^**
**PM_2.5_ µg·m^−3^**	13 657	16±18	7	11	18	10	16	91
**PM_10_ µg·m^−3^**	13 585	17±21	7	12	19	13	18	90
**NO_2_ ppb**	14 739	14±6	10	12	15	4	5	100
**NO ppb**	14 629	9±11	3	5	10	6	10	99
**O_3_ ppb**	14 739	6±5	3	5	8	3	5	100
**CO ppm**	14 522	0.21±0.10	0.15	0.18	0.23	0.04	0.10	98
**Temperature °C**	14 739	21±2	20	21	22	2	2	100

Data are presented as 24-h averages except for ozone (O_3_), which is 8-h maximum. PM_2.5_: particulate matter with aerodynamic diameter <2.5 μm; PM_10_: particulate matter with aerodynamic diameter <10 μm; NO_2_: nitrogen dioxide; NO: nitric oxide; CO: carbon monoxide. ^#^: mean value of the number of valid observations over the number of theoretical observations per participant across the cohort (n_observations_/n_theoretical_).

All pairwise correlation coefficients between air pollutants were relatively low (r<0.4) except for PM_2.5_ and PM_10,_ for which r=0.8 (supplementary table S1). The number of person-days that people left their home without taking the PAM with them was 4083 (28% of total person-days).

### Epidemiological analysis

**FIGURE 1:**
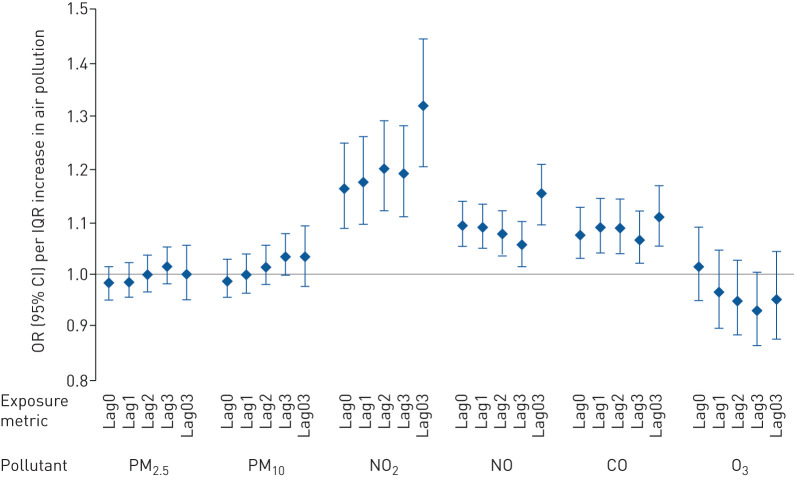
Odds ratios (with 95% confidence intervals) for the occurrence of exacerbation associated with an interquartile range (IQR) increase on the same (Lag0) or previous (Lag1, Lag2 and Lag3) days or the average of the same and previous 3 days (Lag03) for each pollutant. PM_2.5_: particulate matter with aerodynamic diameter <2.5 μm; PM_10_: particulate matter with aerodynamic diameter <10 μm; NO_2_: nitrogen dioxide; NO: nitric oxide; CO: carbon monoxide; O_3_: ozone. Random intercept models adjusted for age, sex, chronic obstructive pulmonary disease severity, Index of Multiple Deprivation rank, inhaled corticosteroid medication use, temperature and time. IQR: PM_2.5_ 10.8 μg·m^−3^, PM_10_ 12.0 μg·m^−3^, NO_2_ 5.2 ppb, NO 7.2 ppb, CO 0.08 ppm and O_3_ 5.0 ppb.

In our analysis for occurrence of an exacerbation, gaseous pollutants were found to be associated with deterioration of COPD patients’ health, increasing the odds of an exacerbation by 16.4% (95% CI 8.6–24.6%), 9.4% (95% CI 5.4–13.6%) and 7.6% (95% CI 3.0–12.4%) per IQR increase in NO_2_, NO and CO, respectively (figure 1). No significant associations were observed for particulate matter in the main or sensitivity analyses that covered the whole study period except for a marginal association for “Lag3”, *i.e.* 3.6% (95% CI 0.0–7.6%) per IQR increase. No significant, but consistently negative, associations were observed for O_3_ in the fully adjusted models.

When lung function was considered with PEF as an indicator, few associations were found to be significant (table 3). We observed an adverse effect of O_3_, *i.e.* 0.5 L·min^−1^ decrease for an IQR increase of the pollutant, and a protective effect for NO which was consistent in the lagged exposure–response analysis.

**TABLE 3:** Associations between personal exposure to air pollutants and peak expiratory flow (PEF) in main and sensitivity analyses

	**Core^#^**	**Full^¶^**		**Lag1 Full**	**Lag2 Full**	**Lag3 Full**	**Lag03 Full**	**Took monitor^+^**
		**Per unit change**	**Per IQR change**					
**PM_2.5_**	−0.045 (−0.306–0.217)	0.004 (−0.021–0.028)	0.038 (−0.226–0.302)	0.055 (−0.212–0.322)	−0.031 (−0.297–0.235)	−0.045 (−0.310–0.220)	0.095 (−0.321–0.511)	−0.080 (−0.379–0.219)
**PM_10_**	−0.085 (−0.354–0.184)	−0.003 (−0.026–0.019)	−0.038 (−0.309–0.233)	0.022 (−0.252–0.296)	−0.077 (−0.351–0.196)	−0.019 (−0.290–0.252)	0.030 (−0.370–0.431)	−0.057 (−0.366–0.252)
**NO_2_**	−0.267 (−0.684–0.150)	0.011 (−0.072–0.094)	0.057 (−0.375–0.490)	−0.146 (−0.583–0.291)	−0.108 (−0.550–0.333)	0.338 (−0.104–0.780)	−0.012 (−0.571–0.546)	0.254 (−0.244–0.751)
**NO**	0.185 (−0.103–0.473)	0.048 (0.008–0.089)*	0.347 (0.055–0.639)*	0.341 (0.042–0.640)*	0.323 (0.021–0.626)*	0.371 (0.068–0.675)*	0.561 (0.163–0.960)*	0.330 (−0.004–0.663)
**CO**	−0.189 (−0.525–0.147)	−0.007 (−0.047–0.033)^§^	−0.058 (−0.397–0.282)	−0.251 (−0.608–0.107)	−0.153 (−0.506–0.200)	−0.112 (−0.463–0.239)	−0.262 (−0.696–0.172)	0.057 (−0.334–0.448)
**O_3_**	−0.134 (−0.543–0.275)	−0.103 (−0.192– −0.013)	−0.517 (−0.969– −0.066)*	−0.438 (−0.905–0.029)	−0.221 (−0.684–0.243)	−0.339 (−0.799–0.122)	−0.372 (−0.869–0.124)	−0.195 (−0.693–0.304)

Data are presented as estimated change in PEF (L·min^−1^) with 95% confidence intervals per interquartile range (IQR) increase, unless otherwise stated. PM_2.5_: particulate matter with aerodynamic diameter <2.5 μm; PM_10_: particulate matter with aerodynamic diameter <10 μm; NO_2_: nitrogen dioxide; NO: nitric oxide; CO: carbon monoxide; O_3_: ozone. IQR: PM_2.5_ 10.8 µg·m^−3^, PM_10_ 12.0 µg·m^−3^, NO_2_ 5.2 ppb, NO 7.2 ppb, CO 0.08 ppm and O_3_ 5.0 ppb. ^#^: includes age, sex, chronic obstructive pulmonary disease severity and each pollutant's same day (Lag0) personal measurement; ^¶^: core model plus Index of Multiple Deprivation rank, inhaled corticosteroid medication use, temperature and time; ^+^: excluding those person-days that participants left their home and forgot to take the portable monitor with them; ^§^: per 0.01 ppm increase. *: statistically significant estimates (p<0.05).

**FIGURE 2:**
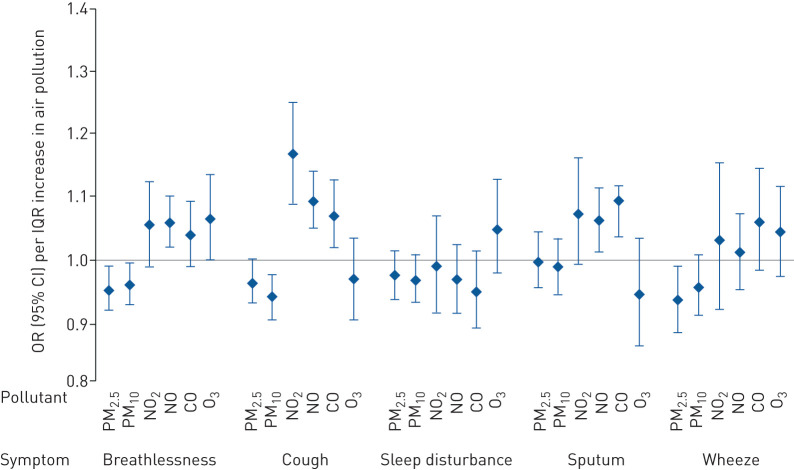
Odds ratios (with 95% confidence intervals) for the occurrence of respiratory symptoms associated with an interquartile range (IQR) increase on the same day (Lag0) for each pollutant. PM_2.5_: particulate matter with aerodynamic diameter <2.5 μm; PM_10_: particulate matter with aerodynamic diameter <10 μm; NO_2_: nitrogen dioxide; NO: nitric oxide; CO: carbon monoxide; O_3_: ozone. Random intercept models adjusted for age, sex, chronic obstructive pulmonary disease severity, Index of Multiple Deprivation rank, inhaled corticosteroid medication use, temperature and time. IQR: PM_2.5_ 10.8 μg·m^−3^, PM_10_ 12.0 μg·m^−3^, NO_2_ 5.2 ppb, NO 7.2 ppb, CO 0.08 ppm and O_3_ 5.0 ppb.

For the self-reported respiratory symptoms, gaseous pollutants were generally found to have a negative impact on participants’ health (figure 2). In particular, breathlessness was associated with NO (OR 1.060 (95% CI 1.019–1.102) per IQR) and O_3_ (OR 1.065 (95% CI 1.000–1.135) per IQR); cough with NO_2_ (OR 1.167 (95% CI 1.088–1.251) per IQR), NO (OR 1.094 (95% CI 1.052–1.139) per IQR) and CO (OR 1.071 (95% CI 1.019–1.125) per IQR); and sputum with NO (OR 1.060 (95% CI 1.012–1.112) per IQR) and CO (OR 1.094 (95% CI 1.035–1.115) per IQR). However, particulate matter was negatively associated with breathlessness, cough and wheeze. Sleep disturbance was not associated with any pollutant.

We observed significant relationships in the winter period subgroup analysis, *i.e.* 0.5% (95% CI 0.1–0.9%), 0.8% (95% CI 0.2–1.3%) and 3.0% (95% CI 0.1–5.8%) decreased risk of exacerbation per unit increase in PM_2.5_, PM_10_ and NO_2_, respectively, but also a negative (adverse) association for NO and a strong negative (adverse) association for O_3_ with OR 1.009 (95% CI 1.001–1.017) and 1.149 (95% CI 1.074–1.229) per unit, respectively (table 4). Particulate matter was found to be statistically significantly associated with a decrease in PEF in the summer period, *i.e.* −0.122 (95% CI −0.186– −0.058) and −0.063 (95% CI −0.113– −0.014) L·min^−1^ per unit increase in PM_2.5_ and PM_10_, respectively. O_3_ was positively and PM_2.5_ was negatively associated with exacerbation in spring. All the other associations were not statistically significant.

**TABLE 4:** Season-specific^#^ estimates for the associations between personal exposure to air pollutants and peak expiratory flow (PEF) and exacerbation

	**Spring**		**Summer**		**Autumn**		**Winter**	
	**PEF**	**Exacerbation**	**PEF**	**Exacerbation**	**PEF**	**Exacerbation**	**PEF**	**Exacerbation**
**PM_2.5_**	−0.011 (−0.059–0.037)	0.986 (0.975–0.997)*	−0.122 (−0.186– −0.058)*	1.010 (1.000–1.021)*	0.012 (−0.041–0.065)	1.000 (0.994–1.005)	0.018 (−0.018–0.053)	0.995 (0.990–0.999)*
**PM_10_**	−0.003 (−0.042–0.035)	1.001 (0.993–1.010)	−0.063 (−0.113– −0.014)*	1.006 (0.998–1.015)	0.006 (−0.048–0.060)	1.000 (0.994–1.005)	0.011 (−0.027–0.049)	0.992 (0.987–0.998)*
**NO_2_**	−0.119 (−0.248–0.011)	1.008 (0.982–1.034)	0.059 (−0.156–0.275)	1.024 (0.976–1.074)	−0.115 (−0.368–0.138)	1.003 (0.964–1.043)	0.063 (−0.092–0.218)	0.970 (0.942–0.999)*
**NO**	0.034 (−0.136–0.205)	1.000 (0.970–1.031)	−0.027 (−0.171–0.117)	1.030 (0.988–1.073)	0.002 (−0.084–0.088)	1.002 (0.990–1.014)	−0.006 (−0.058–0.046)	1.009 (1.001–1.017)*
**CO^¶^**	−0.009 (−0.082–0.064)	0.996 (0.982–1.011)	0.052 (−0.031–0.135)	1.001 (0.985–1.018)	−0.096 (−0.209–0.016)	1.005 (0.988–1.022)	−0.012 (−0.074–0.050)	1.007 (0.995–1.018)
**O_3_**	0.009 (−0.113–0.132)	1.028 (1.004–1.052)*	0.035 (−0.099–0.169)	1.016 (0.987–1.045)	−0.169 (−0.515–0.177)	0.969 (0.913–1.029)	0.008 (−0.411–0.427)	1.149 (1.074–1.229)*

Data are presented as estimated change in PEF (L·min^−1^) or odds ratio for exacerbation with 95% confidence interval by season per unit increase (models adjusted for age, sex, chronic obstructive pulmonary disease severity, Index of Multiple Deprivation rank, inhaled corticosteroid medication use, temperature and time). PM_2.5_: particulate matter with aerodynamic diameter <2.5 μm; PM_10_: particulate matter with aerodynamic diameter <10 μm; NO_2_: nitrogen dioxide; NO: nitric oxide; CO: carbon monoxide; O_3_: ozone. ^#^: spring n=4495, summer n=4507, autumn n=2769, winter n=2969; ^¶^: per 0.01 ppm increase. *: statistically significant estimates (p<0.05).

When we restricted the analysis to person-days in which the patients went outside their home and took the monitor with them (table 3 and supplementary figure S1), only the positive (protective) association between NO and PEF became not statistically significant, and there was a slight decrease in the odds ratios of exacerbation for NO_2_, NO and CO, which did not affect the statistical significance of the estimates.

Analysis of two-pollutant models showed that the statistical significance of the estimates for all pollutants did not change when we adjusted for any of the other co-pollutants except for the effect of O_3_ on exacerbations, which turned null after adjustment (table 5). The odds ratio of exacerbation for NO_2_, NO and CO decreased in two-pollutant models, showing a potential confounding effect between these gaseous pollutants. The negative association of O_3_ and PEF remained unchanged. When we performed analysis of season-specific two-pollutant models, the findings for each season were almost identical to table 4 (results not shown).

**TABLE 5:** Two-pollutant models for the associations between personal exposure to air pollutants and peak expiratory flow (PEF) and exacerbation

	**Unadjusted**	**PM_2.5_-adjusted**	**PM_10_-adjusted**	**NO_2_-adjusted**	**NO-adjusted**	**CO-adjusted**	**O_3_-adjusted**
**PEF**							
PM_2.5_	0.038 (−0.226–0.302)			0.036 (−0.228–0.300)	−0.010 (−0.275–0.256)	0.041 (−0.226–0.307)	0.033 (−0.231–0.297)
PM_10_	−0.038 (−0.309–0.233)			−0.040 (−0.311–0.232)	−0.077 (−0.349–0.195)	−0.039 (−0.312–0.234)	−0.039 (−0.310–0.232)
NO_2_	0.057 (−0.375–0.490)	0.063 (−0.378–0.505)	0.080 (−0.363–0.523)		−0.087 (−0.537–0.364)	0.063 (−0.390–0.517)	0.022 (−0.412–0.456)
NO	0.347 (0.055–0.639)*	0.429 (0.116–0.742)*	0.438 (0.126–0.750)*	0.360 (0.061–0.659)*		0.402 (0.095–0.709)*	0.339 (0.047–0.630)*
CO	−0.058 (−0.397–0.282)	−0.036 (−0.428–0.356)	−0.013 (−0.406–0.380)	−0.070 (−0.420–0.281)	−0.202 (−0.560–0.155)		−0.097 (−0.438–0.245)
O_3_	−0.517 (−0.969– −0.066)*	−0.551 (−1.015– −0.087)*	−0.551 (−1.018– −0.083)*	−0.516 (−0.968– −0.063)*	−0.498 (−0.950– −0.046)*	−0.528 (−0.985– −0.071)*	
**Exacerbation**							
PM_2.5_	0.983 (0.953–1.014)			0.982 (0.951–1.013)	0.975 (0.944–1.006)	0.977 (0.946–1.008)	0.983 (0.953–1.015)
PM_10_	0.992 (0.957–1.028)			0.994 (0.958–1.030)	0.984 (0.949–1.021)	0.985 (0.950–1.022)	0.992 (0.957–1.029)
NO_2_	1.164 (1.086–1.246)*	1.158 (1.080–1.242)*	1.157 (1.079–1.240)*		1.126 (1.048–1.210)*	1.138 (1.060–1.221)*	1.167 (1.089–1.250)
NO	1.094 (1.054–1.136)*	1.084 (1.043–1.128)*	1.082 (1.040–1.125)*	1.077 (1.036–1.119)*		1.080 (1.038–1.124)*	1.095 (1.055–1.137)*
CO	1.076 (1.030–1.124)*	1.067 (1.018–1.118)*	1.065 (1.016–1.116)*	1.055 (1.011–1.101)*	1.044 (1.000–1.090)*		1.079 (1.033–1.128)*
O_3_	1.017 (0.950–1.089)	1.036 (0.967–1.110)	1.037 (0.968–1.111)	1.031 (0.964–1.103)	1.026 (0.959–1.098)	1.042 (0.973–1.114)	

Data are presented as estimated change in PEF (L·min^−1^) or odds ratio for exacerbation with 95% confidence interval per interquartile range (IQR) increase (models adjusted for age, sex, chronic obstructive pulmonary disease severity, Index of Multiple Deprivation rank, inhaled corticosteroid medication use, temperature and time). PM_2.5_: particulate matter with aerodynamic diameter <2.5 μm; PM_10_: particulate matter with aerodynamic diameter <10 μm; NO_2_: nitrogen dioxide; NO: nitric oxide; CO: carbon monoxide; O_3_: ozone. IQR: PM_2.5_ 10.8 µg·m^−3^, PM_10_ 12.0 µg·m^−3^, NO_2_ 5.2 ppb, NO 7.2 ppb, CO 0.08 ppm and O_3_ 5.0 ppb. *: statistically significant estimates (p<0.05).

Effect modification by COPD severity was observed for the NO_2_ and CO and PEF associations, and the NO, CO and O_3_ and exacerbation associations (supplementary table S6). Except for the latter, all these significant interaction terms were found to support adverse effects for the mild/moderate severity group and protective effects for the severe/very severe group. However, not all associations were statistically significant. When ambient measurements were used as exposure metrics, we found that statistically significant associations between exacerbations and personal exposure to NO and CO turned null, while the NO_2_ odds ratio remained practically the same (supplementary table S7). For PEF, we observed some changes in the statistical significance of the estimates (PM_2.5_ and NO_2_ effects were significant in the models with ambient concentrations, NO with personal exposures); however, all the effects were contrary to the expected direction (*i.e.* more exposure to ambient PM_2.5_ and NO_2_ was associated with an increase in PEF). The O_3_ association was almost doubled.

## Discussion

Improving our knowledge on the associations between environmental stress and COPD symptoms and exacerbations has the potential to facilitate better patient care by predicting when patients are at increased risk of exacerbation. These associations could also be used to warn patients of periods of high risk and prevent exacerbations as a result, thus improving quality of life and reducing mortality.

In this study we investigated the associations between COPD patients’ respiratory health and their personal exposure to air pollution, including ambient (outdoor) and indoor environments. A unique database for 115 participants followed intensively for up to 6 months was utilised. Personal exposures to various air pollutants, along with health data and information about potential confounders, were collected, resulting in more than 14 700 person-days of observations. We observed consistent positive (adverse) associations between respiratory symptoms, *i.e.* exacerbations, breathlessness, cough and sputum, and gaseous pollutants such as NO_2_, NO and CO. O_3_ was negatively associated with PEF. Particulate matter was not found to have associations with any adverse effects on patients’ health.

We assessed the robustness of our findings with various sensitivity analyses. We fitted two-pollutant models to account for the potential confounding effect between pollutants, we excluded from the analysis person-days that the patients went out without their monitor and we checked whether season was an effect modifier. A large positive association between O_3_ and exacerbation in winter can be explained by a combination of uniformly low winter-time O_3_ concentrations indoors [21] and the inverse relationship with NO through titration [22]. Additionally, some apparently protective effects were observed for particulate matter and NO_2_ during the same season. These findings could not be explained by two-pollutant models, in which the estimates remained unchanged.

We also compared the health effect estimates when ambient (central monitor) measurements were used instead of personal exposures. Some differences were observed in the estimates, both qualitatively and quantitatively, but we expected this might be the case. Epidemiological studies typically use central monitoring site data or, more commonly in recent years, ambient models to estimate an individual's personal exposure to ambient air pollution, whereas we measured total personal exposure directly. Therefore, this comparison must be interpreted with caution: one method assesses the impact of ambient air pollution exposure, the other assesses total air pollution exposure. Furthermore, such studies use ambient concentrations as a proxy for personal exposure. In most cases, this will represent an overestimate because <100% of ambient pollution typically infiltrates into buildings, where individuals spend most of their time. Therefore, a unit increase represents that proxy measure, whereas personal measurements are not a proxy. Thus, epidemiological estimates per unit increase cannot be compared directly using the two methods and, therefore, we expressed associations per IQR increase in this study. We are currently developing statistical methods to allow a direct comparison of exposure methods, including separation of indoor and outdoor source exposures, which will facilitate estimates of bias and the value of absolute *versus* proxy measures of exposure. It should be noted that our subjects were relatively old, had a chronic respiratory condition and probably spent more time at home compared with other groups of the same population.

Despite that, our findings are in close agreement with Peacock *et al*. [23] who conducted a large panel study with COPD patients in London. Results were similar for the associations between matching pollutants and health outcomes, although their study only assessed exposure to ambient pollutants. Similarly, absence of significant associations between particle mass and PEF has been reported in other studies with COPD patients [24–26]. Chamber studies, which measure absolute exposure, may be more appropriate for comparison with our results. In the two “Oxford Street” natural chamber studies [11, 27], consistent adverse associations in asthma and COPD patients were found between respiratory functions and ultrafine particulate matter (a pollutant not measured in our study), but not PM_2.5_. Laboratory chamber study results are mixed for all pollutants [28–30].

The COPE study benefited from the plethora of personal exposure and health outcome measurements obtained from a relatively large number of participants. Similar previous personal exposure studies have either focused on one or two pollutants only [31, 32] and/or limited their follow-up to a few days or weeks [25, 33]. Using monitors designed specifically for this study, we were able to collect measurements for six different pollutants. In addition, we reached a median follow-up of 145 days per participant, covering a long, representative period of mixed seasons.

However, the main strength of the COPE study is the collection of sustained personal exposure measurements which, even though it was a demanding task for the patients, had high levels of compliance. Anecdotal evidence suggests that this is because COPD patients are used to long-term clinical monitoring and could see direct benefit to themselves. Previous studies have used datasets of similar size, but instead of personal exposures they used ambient measurements of pollution [23, 34]. This exposure metric can differ substantially from the actual exposures of the patients for multiple reasons, including exposure to both ambient and indoor sources of pollution, variable infiltration factors of buildings, time–activity patterns of the individuals, concentrations in different micro-environments, and spatial aggregation [35]. This may have resulted in biased epidemiological estimates due to exposure measurement error [36–38]. In this study, we collected personal exposure data to accurately represent the quality of air that the participants breathed, rather than a proxy estimate.

Our study is subject to some limitations. First, the COPE study was conducted in London, which, even though it is a highly ethnically diverse metropolitan city, cannot provide a representative sample of COPD patients worldwide. Despite this, the findings seem to be in agreement with some studies from other locations using different methodologies [26, 33, 39].

Because of the length and the demands of this study, some participants did not always carry the monitor with them when they left home. However, our results were unchanged by restricting our analysis to those that did take the monitor with them. It should be noted that COPD patients are less mobile and more used to carrying/using medical equipment than other population groups.

Finally, we have performed many ancillary analyses, some of which may have resulted in statistically significant associations by chance. However, finding consistent associations for gaseous pollutants for various health outcomes makes this unlikely. However, we observed some unexpected negative associations between particulate matter exposures and respiratory symptoms which were difficult to explain biologically. A potential reason might be the use of total personal exposure as the metric of interest, as, even though it reflects what patients actually breathe, indoor- and outdoor-generated pollution comprise different sources, composition and behaviours [35]. False-positive associations might be the result of treating pollution from different sources equally, without accounting for potentially different health effects [40]. This issue, along with the impact of mobility in the exposure estimates, will be further investigated by separating total personal exposure into indoor- and outdoor-generated pollution and re-applying the models in future work. This partition is very important for policy making as regulations and mitigation actions for indoor pollution are very different from those for ambient air due to their contrasting sources, *i.e.* traffic, industrial and agricultural emissions *versus* domestic cooking, heating and cleaning emissions. Such partitioning will also facilitate identification of dominant sources of each pollutant in the indoor and outdoor environment [15].

### Conclusions

This study demonstrated that it is feasible to gather robust multipollutant personal exposure measurements over extended periods in a cohort of COPD patients. By utilising personal measurements, total exposure can be assessed at unprecedented detail. Significant associations were found between an individual's exposure to gaseous pollutants and their respiratory health, but not particulate pollutants. While this finding is important in confirming links between personal pollutant exposure and patient health, further work is required to identify which sources drive this association and whether these operate indoors or outdoors.

## Supplementary material

10.1183/13993003.03432-2020.Supp1 **Please note:** supplementary material is not edited by the Editorial Office, and is uploaded as it has been supplied by the author.
Supplementary material ERJ-03432-2020.SUPPLEMENT

## Shareable PDF

10.1183/13993003.03432-2020.Shareable1 This one-page PDF can be shared freely online.
Shareable PDF ERJ-03432-2020.Shareable

## References

[1] World Health Organization. Disease Burden and Mortality Estimates, Cause-specific Mortality, 2000–2016. Geneva, WHO, 2016.

[2] López-Campos JL, Tan W, Soriano JB. Global burden of COPD. Respirology 2016; 21: 14–23. doi:10.1111/resp.12660 26494423

[3] Anzueto A. Impact of exacerbations on COPD. Eur Respir Rev 2010; 19: 113–118. doi:10.1183/09059180.00002610 20956179 PMC9682573

[4] Anderson HR, Spix C, Medina S, et al. Air pollution and daily admissions for chronic obstructive pulmonary disease in 6 European cities: results from the APHEA project. Eur Respir J 1997; 10: 1064–1071. doi:10.1183/09031936.97.10051064 9163648

[5] Sunyer J, Saez M, Murillo C, et al. Air pollution and emergency room admissions for chronic obstructive pulmonary disease: a 5-year study. Am J Epidemiol 1993; 137: 701–705. doi:10.1093/oxfordjournals.aje.a116730 8484361

[6] Halonen JI, Lanki T, Yli-Tuomi T, et al. Urban air pollution, and asthma and COPD hospital emergency room visits. Thorax 2008; 63: 635–641. doi:10.1136/thx.2007.091371 18267984

[7] Moore E, Chatzidiakou L, Kuku MO, et al. Global associations between air pollutants and chronic obstructive pulmonary disease hospitalizations. A systematic review. Ann Am Thorac Soc 2016; 13: 1814–1827. doi:10.1513/AnnalsATS.201603-196CME 27314857 PMC5122486

[8] Arbex MA, de Souza Conceicao GM, Cendon SP, et al. Urban air pollution and chronic obstructive pulmonary disease-related emergency department visits. J Epidemiol Community Health 2009; 63: 777–783. doi:10.1136/jech.2008.078360 19468016

[9] Butland BK, Samoli E, Atkinson RW, et al. Measurement error in a multi-level analysis of air pollution and health: a simulation study. Environ Health 2019; 18: 13. doi:10.1186/s12940-018-0432-8 30764837 PMC6376751

[10] Chan AT, Chung MW. Indoor–outdoor air quality relationships in vehicle: effect of driving environment and ventilation modes. Atmos Environ 2003; 37: 3795–3808. doi:10.1016/S1352-2310(03)00466-7

[11] McCreanor J, Cullinan P, Nieuwenhuijsen MJ, et al. Respiratory effects of exposure to diesel traffic in persons with asthma. N Engl J Med 2007; 357: 2348–2358. doi:10.1056/NEJMoa071535 18057337

[12] Mead M, Popoola O, Stewart G, et al. The use of electrochemical sensors for monitoring urban air quality in low-cost, high-density networks. Atmos Environ 2013; 70: 186–203. doi:10.1016/j.atmosenv.2012.11.060

[13] Moore E, Chatzidiakou L, Jones RL, et al. Linking e-health records, patient-reported symptoms and environmental exposure data to characterise and model COPD exacerbations: protocol for the COPE study. BMJ Open 2016; 6: e011330. doi:10.1136/bmjopen-2016-011330 PMC4947745 27412104

[14] Quint JK, Moore E, Lewis A, et al. Recruitment of patients with Chronic Obstructive Pulmonary Disease (COPD) from the Clinical Practice Research Datalink (CPRD) for research. NPJ Prim Care Respir Med 2018; 28: 21. doi:10.1038/s41533-018-0089-3 29921879 PMC6008416

[15] Chatzidiakou L, Krause A, Popoola OA, et al. Characterising low-cost sensors in highly portable platforms to quantify personal exposure in diverse environments. Atmos Meas Tech 2019; 12: 4643–4657. doi:10.5194/amt-12-4643-2019 31534556 PMC6751078

[16] McNicholas WT, Verbraecken J, Marin JM. Sleep disorders in COPD: the forgotten dimension. Eur Respir Rev 2013; 22: 365–375. doi:10.1183/09059180.00003213 23997063 PMC9487346

[17] Wedzicha JA, Seemungal TA. COPD exacerbations: defining their cause and prevention. Lancet 2007; 370: 786–796. doi:10.1016/S0140-6736(07)61382-8 17765528 PMC7134993

[18] Ministry of Housing, Communities and Local Government. English indices of deprivation 2015. 2015. http://imd-by-postcode.opendatacommunities.org/imd/2015. Date accessed: December 31, 2019.

[19] Bloemsma LD, Hoek G, Smit LA. Panel studies of air pollution in patients with COPD: systematic review and meta-analysis. Environ Res 2016; 151: 458–468. doi:10.1016/j.envres.2016.08.018 27565881

[20] Janes H, Sheppard L, Shepherd K. Statistical analysis of air pollution panel studies: an illustration. Ann Epidemiol 2008; 18: 792–802. doi:10.1016/j.annepidem.2008.06.004 18922395

[21] Salonen H, Salthammer T, Morawska L. Human exposure to ozone in school and office indoor environments. Environ Int 2018; 119: 503–514. doi:10.1016/j.envint.2018.07.012 30053738

[22] World Health Organization. Review of Evidence on Health Aspects of Air Pollution – REVIHAAP Project: Final Technical Report. Geneva, WHO, 2013.

[23] Peacock JL, Anderson HR, Bremner SA, et al. Outdoor air pollution and respiratory health in patients with COPD. Thorax 2011; 66: 591–596. doi:10.1136/thx.2010.155358 21459856

[24] Trenga CA, Sullivan JH, Schildcrout JS, et al. Effect of particulate air pollution on lung function in adult and pediatric subjects in a Seattle panel study. Chest 2006; 129: 1614–1622. doi:10.1378/chest.129.6.1614 16778283

[25] De Hartog J, Ayres J, Karakatsani A, et al. Lung function and indicators of exposure to indoor and outdoor particulate matter among asthma and COPD patients. Occup Environ Med 2010; 67: 2–10. doi:10.1136/oem.2008.040857 19736175

[26] Ni Y, Wu S, Ji W, et al. The exposure metric choices have significant impact on the association between short-term exposure to outdoor particulate matter and changes in lung function: findings from a panel study in chronic obstructive pulmonary disease patients. Sci Total Environ 2016; 542: 264–270. doi:10.1016/j.scitotenv.2015.10.114 26519586

[27] Sinharay R, Gong J, Barratt B, et al. Respiratory and cardiovascular responses to walking down a traffic-polluted road compared with walking in a traffic-free area in participants aged 60 years and older with chronic lung or heart disease and age-matched healthy controls: a randomised, crossover study. Lancet 2018; 391: 339–349. doi:10.1016/S0140-6736(17)32643-0 29221643 PMC5803182

[28] US Environmental Protection Agency. Integrated Science Assessment (ISA) for Particulate Matter (Final Report). EPA/600/R-19/188. Washington, US EPA, 2019.

[29] US Environmental Protection Agency. Integrated Science Assessment (ISA) for Ozone and Related Photochemical Oxidants (Final Report). EPA/600/R-20/012. Washington, US EPA, 2020.

[30] US Environmental Protection Agency. Integrated Science Assessment (ISA) For Oxides of Nitrogen – Health Criteria (Final Report). EPA/600/R-15/068. Washington, US EPA, 2016.

[31] Alahmari AD, Mackay AJ, Patel AR, et al. Influence of weather and atmospheric pollution on physical activity in patients with COPD. Respir Res 2015; 16: 71. doi:10.1186/s12931-015-0229-z 26071400 PMC4470337

[32] Cortez-Lugo M, Ramirez-Aguilar M, Perez-Padilla R, et al. Effect of personal exposure to PM_2.5_ on respiratory health in a Mexican panel of patients with COPD. Int J Environ Res Public Health 2015; 12: 10635–10647. doi:10.3390/ijerph120910635 26343703 PMC4586633

[33] Higgins B, Francis H, Yates C, et al. Environmental exposure to air pollution and allergens and peak flow changes. Eur Respir J 2000; 16: 61–66. doi:10.1034/j.1399-3003.2000.16a11.x 10933086

[34] Karakatsani A, Analitis A, Perifanou D, et al. Particulate matter air pollution and respiratory symptoms in individuals having either asthma or chronic obstructive pulmonary disease: a European multicentre panel study. Environ Health 2012; 11: 75. doi:10.1186/1476-069X-11-75 23039312 PMC3509003

[35] Evangelopoulos D, Katsouyanni K, Keogh RH, et al. PM_2.5_ and NO_2_ exposure errors using proxy measures, including derived personal exposure from outdoor sources: a systematic review and meta-analysis. Environ Int 2020; 137: 105500. doi:10.1016/j.envint.2020.105500 32018132

[36] Armstrong BG. Effect of measurement error on epidemiological studies of environmental and occupational exposures. Occup Environ Med 1998; 55: 651–656. doi:10.1136/oem.55.10.651 9930084 PMC1757516

[37] Zeger SL, Thomas D, Dominici F, et al. Exposure measurement error in time-series studies of air pollution: concepts and consequences. Environ Health Perspect 2000; 108: 419–426. doi:10.1289/ehp.00108419 10811568 PMC1638034

[38] Butland BK, Samoli E, Atkinson RW, et al. Comparing the performance of air pollution models for nitrogen dioxide and ozone in the context of a multilevel epidemiological analysis. Environ Epidemiol 2020; 4: e093. doi:10.1097/EE9.0000000000000093 32656488 PMC7319188

[39] Sun XW, Chen PL, Ren L, et al. The cumulative effect of air pollutants on the acute exacerbation of COPD in Shanghai, China. Sci Total Environ 2018; 622: 875–881. doi:10.1016/j.scitotenv.2017.12.042 29227938

[40] Ebelt ST, Wilson WE, Brauer M. Exposure to ambient and nonambient components of particulate matter – a comparison of health effects. Epidemiology 2005; 16: 396–405. doi:10.1097/01.ede.0000158918.57071.3e 15824557

