# Supplementary material for: Personal exposure to air pollution and respiratory health of COPD patients in London
Source: Eur Respir J. 2021 Jul 15;58(1):2003432. doi: 10.1183/13993003.03432-2020 (PMC8290182; doi:10.1183/13993003.03432-2020)
Supplement: Supplementary file 1 [file ERJ-03432-2020.SUPPLEMENT.pdf]

Table S1 – Pearson correlation coefficients between any pair of personal exposure to air pollutants based on 14,740 person-days of follow-up. All exposures are expressed as 24-hour means, except for O_3 which is 8-hour maximum.

	$PM_{2.5}$	PM_{10}	NO_2	NO	CO	O_3
**$PM_{2.5}$**	1					
**PM_{10}**	0.816	1				
**NO_2**	0.110	0.118	1			
**NO**	0.235	0.152	0.212	1		
**CO**	0.213	0.165	0.241	0.399	1	
**O_3**	-0.086	-0.031	0.067	-0.145	-0.157	1

All results are statistically significant at significance level of <0.001.

Table S2 - Descriptive statistics for personal exposure to air pollution, expressed as 24-hour averages, except for O3 which is 8-hour maximum by season. N: number of valid measurements.

Personal Exposure	Season	N	Mean	SD	25th %ile	Median	75th %ile
**$PM_{2.5}$ (µg/m³)**	Spring	4,223	15.0	13.4	7.6	11.5	17.6
	Summer	4,152	10.4	10.3	5.6	8.1	11.9
	Autumn	2,517	17.7	20.7	8.8	13.3	19.4
	Winter	2,765	23.5	25.8	11.1	17.0	26.8
**PM_{10} (µg/m³)**	Spring	4,200	16.9	21.3	6.1	11.5	18.5
	Summer	4,103	11.2	14.0	4.3	8.6	13.5
	Autumn	2,517	18.4	20.5	10.1	14.2	20.2
	Winter	2,765	23.6	25.1	11.3	17.3	26.9
**NO_2 (ppb)**	Spring	4,495	14.6	6.7	10.5	13.0	16.3
	Summer	4,506	11.7	3.4	9.4	10.7	13.0
	Autumn	2,769	13.8	5.5	10.6	12.6	15.3
	Winter	2,969	15.8	8.5	11.2	13.7	17.3
**NO (ppb)**	Spring	4,456	6.1	4.8	3.0	4.7	7.3
	Summer	4,463	4.5	5.2	2.3	3.5	5.2
	Autumn	2,753	12.6	13.1	4.6	8.6	16.2
	Winter	2,957	16.9	17.0	6.0	11.5	21.4
**O_3 (ppb)**	Spring	4,495	6.6	5.4	3.7	5.4	8.3
	Summer	4,506	8.5	6.0	4.7	7.0	10.6
	Autumn	2,769	5.0	4.1	2.5	4.0	6.1
	Winter	2,969	4.5	3.4	2.2	3.4	5.7
**CO (ppm)**	Spring	4,425	0.19	0.09	0.15	0.17	0.21
	Summer	4,429	0.18	0.07	0.14	0.16	0.19
	Autumn	2,724	0.23	0.09	0.17	0.21	0.26
	Winter	2,944	0.25	0.14	0.18	0.23	0.30

Table S3 - Descriptive statistics for ambient measurements of air pollution as measured from the nearest monitor of the London Air Quality Network (LAQN), expressed as 24-hour averages, except for O3 which is 8-hour maximum. N obs: number of valid observations.

Exposure	N obs	Mean	SD	25th %ile	Median	75th %ile
**$PM_{2.5}$ (µg/m³)**	13,646	12.4	10.4	6.3	8.7	14.3
**PM_{10} (µg/m³)**	13,596	19.1	11.1	12.0	15.4	22.7
**NO_2 (ppb)**	14,739	17.2	9.3	10.6	15.6	21.5
**NO (ppb)**	14,630	11.0	26.6	1.7	3.1	7.3
**O_3 (ppb)**	14,647	29.4	12.3	22.1	0.16	0.21
**CO (ppm)**	14,289	0.20	0.14	0.13	30.3	37.0

The measurements are from the nearest monitor of the London Air Quality Network (http://www.londonair.org.uk/LondonAir/Default.aspx).

Table S4 - Associations between personal exposure to air pollutants and exacerbation in main and sensitivity analyses. In bold are the statistically significant estimates.

	OR for exacerbation with 95% CI per interquartile range increase (except otherwise stated)							
	**Core[a]**	**Full[b]**		**Lag1 Full**	**Lag2 Full**	**Lag3 Full**	**Lag03 Full**	**Took monitor[c]**
		**per unit change**	**per IQR change**					
**$PM_{2.5}$**	0.998 (0.967, 1.029)	0.998 (0.995, 1.001)	0.983 (0.953, 1.014)	0.990 (0.959, 1.022)	1.001 (0.969, 1.034)	1.016 (0.982, 1.051)	1.001 (0.952, 1.054)	0.978 (0.944, 1.014)
**PM_{10}**	1.008 (0.973, 1.044)	0.999 (0.996, 1.002)	0.992 (0.957, 1.028)	1.002 (0.965, 1.040)	1.018 (0.980, 1.057)	1.036 (0.997, 1.076)	1.032 (0.977, 1.090)	0.983 (0.943, 1.025)
**NO_2**	**1.227 (1.149, 1.311)**	**1.029 (1.016, 1.043)**	**1.164 (1.086, 1.246)**	**1.175 (1.096, 1.259)**	**1.201 (1.120, 1.288)**	**1.192 (1.111, 1.279)**	**1.319 (1.206, 1.444)**	**1.156 (1.071, 1.248)**
**NO**	**1.119 (1.079, 1.161)**	**1.013 (1.007, 1.018)**	**1.094 (1.054, 1.136)**	**1.089 (1.048, 1.132)**	**1.077 (1.036, 1.120)**	**1.057 (1.017, 1.099)**	**1.150 (1.093, 1.210)**	**1.076 (1.032, 1.122)**
**CO**	1.014 (0.966, 1.046)	**1.009 (1.003, 1.014)[d]**	**1.076 (1.030, 1.124)**	**1.091 (1.040, 1.145)**	**1.090 (1.040, 1.142)**	**1.068 (1.021, 1.117)**	**1.110 (1.055, 1.169)**	**1.061 (1.009, 1.117)**
**O_3**	**0.918 (0.861, 0.979)**	1.003 (0.990, 1.017)	1.017 (0.950, 1.089)	0.970 (0.900, 1.045)	0.953 (0.885, 1.026)	0.933 (0.866, 1.005)	0.956 (0.878, 1.041)	1.032 (0.957, 1.112)

[a]Includes: age, sex, COPD severity and each pollutant's same day (Lag0) personal measurement.
[b]Core model plus IMD rank, ICS medication use, temperature and time.
[c]Excluding those person-days that participants left their house and forgot to take the portable monitor.
[d]Per 0.01 ppm increase.

Figure S1 - Estimated change in PEF (L/min) associated with an IQR increase on the same (Lag0) or previous (Lag1, Lag2, Lag3) days or the average of the same and three previous days (Lag03) for personal exposure to each pollutant. Random intercept models adjusted for age, sex, COPD severity, IMD rank, ICS medication use, temperature and time.

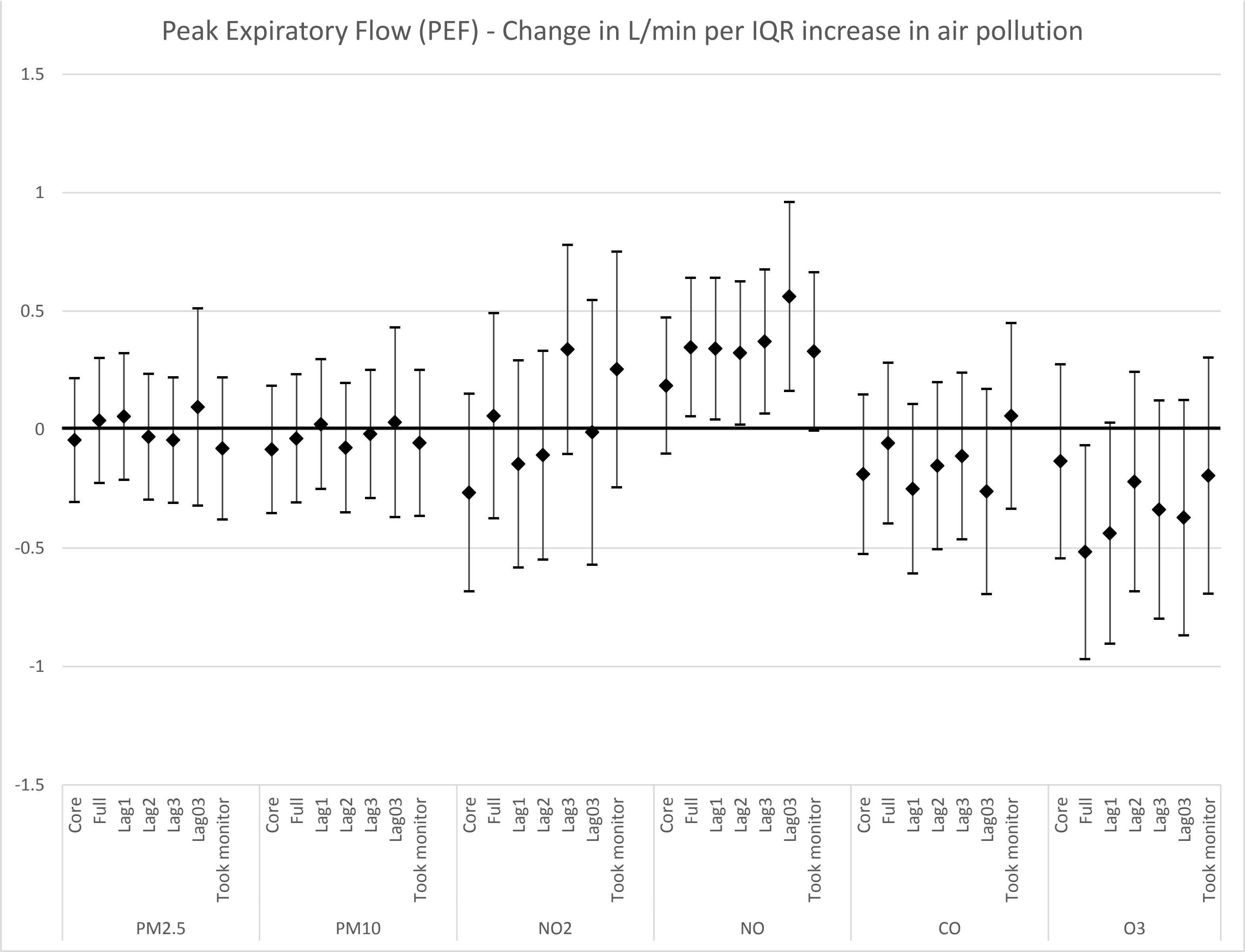

Table S5 - Associations between personal exposure to air pollutants and various respiratory symptoms. In bold are the statistically significant estimates.

	OR for respiratory symptoms with 95% CI[a]									
	Breathlessness		Cough		Sleep disturbance		Sputum		Wheeze	
	per unit change	per IQR change	per unit change	per IQR change	per unit change	per IQR change	per unit change	per IQR change	per unit change	per IQR change
**$PM_{2.5}$**	**0.996 (0.992, 0.999)**	**0.953 (0.919, 0.989)**	**0.996 (0.993, 1.000)**	**0.963 (0.929, 0.998)**	0.998 (0.994, 1.001)	0.974 (0.935, 1.014)	1.000 (0.996, 1.004)	0.997 (0.955, 1.042)	**0.994 (0.989, 0.999)**	**0.935 (0.883, 0.990)**
**PM_{10}**	**0.997 (0.994, 0.999)**	**0.959 (0.926, 0.994)**	**0.995 (0.992, 0.998)**	**0.939 (0.903, 0.976)**	0.997 (0.994, 1.001)	0.968 (0.932, 1.006)	0.999 (0.995, 1.003)	0.988 (0.944, 1.033)	0.996 (0.992, 1.000)	0.957 (0.911, 1.005)
**NO_2**	1.010 (0.998, 1.022)	1.053 (0.988, 1.122)	**1.030 (1.016, 1.044)**	**1.167 (1.088, 1.251)**	0.998 (0.983, 1.013)	0.989 (0.914, 1.069)	1.014 (0.999, 1.029)	1.073 (0.992, 1.161)	1.006 (0.985, 1.027)	1.030 (0.922, 1.152)
**NO**	**1.008 (1.003, 1.014)**	**1.060 (1.019, 1.102)**	**1.013 (1.007, 1.018)**	**1.094 (1.052, 1.139)**	0.995 (0.988, 1.003)	0.967 (0.914, 1.022)	**1.008 (1.002, 1.015)**	**1.060 (1.012, 1.112)**	1.001 (0.993, 1.010)	1.010 (0.952, 1.072)
**CO**	1.004 (0.999, 1.010)[b]	1.038 (0.988, 1.091)	**1.008 (1.002, 1.014)**[b]	**1.071 (1.019, 1.125)**	0.994 (0.986, 1.001)[b]	0.948 (0.890, 1.010)	**1.011 (1.004, 1.017)**[b]	**1.094 (1.035, 1.115)**	1.007 (0.998, 1.016)[b]	1.060 (0.982, 1.144)
**O_3**	**1.013 (1.000, 1.025)**	**1.065 (1.000, 1.135)**	0.993 (0.980, 1.007)	0.967 (0.904, 1.033)	1.010 (0.996, 1.024)	1.050 (0.979, 1.126)	0.989 (0.971, 1.007)	0.944 (0.862, 1.034)	1.008 (0.994, 1.022)	1.042 (0.972, 1.116)

[a]Models adjusted for age, sex, COPD severity, IMD rank, ICS medication use, temperature and time.
[b]Per 0.01 ppm increase.

Table S6 –Associations between personal exposure to air pollutants and peak expiratory flow and exacerbation by COPD severity. In bold are the statistically significant interaction term at the 0.05 level.

	Estimated change in PEF (L/min) or OR for exacerbation with 95% CI by COPD severity per unit increase[a]			
	PEF		Exacerbation	
	Mild or Moderate COPD	Severe or Very severe COPD	Mild or Moderate COPD	Severe or Very severe COPD
**$PM_{2.5}$**	0.006 (-0.022, 0.034)	-0.004 (-0.054, 0.046)	0.998 (0.995, 1.001)	1.000 (0.994, 1.006)
**PM_{10}**	-0.002 (-0.028, 0.024)	-0.007 (-0.051, 0.037)	0.998 (0.995, 1.002)	1.002 (0.996, 1.008)
**NO_2**	**-0.090 (-0.189, 0.010)**	**0.229 (0.084, 0.374)**	1.037 (1.019, 1.055)	1.020 (1.001, 1.040)
**NO**	0.029 (-0.019, 0.076)	0.099 (0.023, 0.175)	**1.025 (1.018, 1.032)**	**0.990 (0.980, 1.000)**
**CO**	**-0.034 (-0.079, 0.012)[b]**	**0.086 (0.012, 0.017)[b]**	**1.018 (1.010, 1.025)[b]**	**0.993 (0.983, 1.002)[b]**
**O_3**	-0.099 (-0.206, 0.009)	-0.110 (-0.253, 0.031)	**0.933 (0.911, 0.955)**	**1.051 (1.033, 1.070)**

[a]Models adjusted for age, sex, COPD severity, IMD rank, ICS medication use, temperature and time.
[b]Per 0.01 ppm increase.

Table S7 - Associations between air pollutants and peak expiratory flow and exacerbation using personal exposures or ambient measurements as measured from the nearest monitor as exposure metrics. In bold are the statistically significant estimates.

	PEF – Change per IQR increase (95% CI)[a]		Exacerbation – OR per IQR increase (95% CI)[a]	
	**Ambient[b]**	**Personal[c]**	**Ambient[b]**	**Personal[c]**
**$PM_{2.5}$**	**0.327 (0.001, 0.654)**	0.038 (-0.226, 0.302)	1.017 (0.975, 1.061)	0.983 (0.953, 1.014)
**PM_{10}**	0.329 (-0.077, 0.734)	-0.038 (-0.309, 0.233)	1.032 (0.979, 1.088)	0.992 (0.957, 1.028)
**NO_2**	**0.669 (0.156, 1.182)**	0.057 (-0.375, 0.490)	**1.159 (1.084, 1.240)**	**1.164 (1.086, 1.246)**
**NO**	0.074 (-0.014, 0.162)	**0.347 (0.055, 0.639)**	1.007 (0.996, 1.018)	**1.094 (1.054, 1.136)**
**CO**	0.195 (-0.059, 0.449)	-0.058 (-0.397, 0.282)	1.027 (0.994, 1.061)	**1.076 (1.030, 1.124)**
**O_3**	**-1.019 (-1.591, -0.447)**	**-0.517 (-0.969, -0.066)**	0.973 (0.900, 1.052)	1.017 (0.950, 1.089)

[a]Models adjusted for age, sex, COPD severity, IMD rank, ICS medication use, temperature and time.
[b]Ambient measurements IQRs: $PM_{2.5}$ = 8.0 µg/m^3, PM_{10} = 10.7 µg/m^3, NO_2 = 10.9 ppb, NO = 5.6 ppb, CO = 0.08 ppm, O_3 = 15.0 ppb.
[c]Personal exposures IQRs: $PM_{2.5}$ = 10.8 µg/m^3, PM_{10} = 12.0 µg/m^3, NO_2 = 5.2 ppb, NO = 7.2 ppb, CO = 0.08 ppm, O_3 = 5.0 ppb.
