# Supplementary material for: Personal exposure to air pollution and respiratory health of COPD patients in London
Source: Eur Respir J. 2021 Jul 15;58(1):2003432. doi: 10.1183/13993003.03432-2020 (PMC8290182; doi:10.1183/13993003.03432-2020)
Supplement: Supplementary file 2 [file ERJ-03432-2020.Shareable.pdf]

SHAREABLE PDF

Personal exposure to air pollution and respiratory health of COPD patients in London

Dimitris Evangelopoulos [1,2], Lia Chatzidiakou [3], Heather Walton [1,2], Klea Katsouyanni [1,4], Frank J. Kelly [1,2], Jennifer K. Quint [5], Roderic L. Jones [3] and Benjamin Barratt [1,2]

Affiliations: [1]Environmental Research Group, MRC Centre for Environment and Health, Imperial College London, London, UK. [2]National Institute for Health Research Health Protection Research Unit in Environmental Exposures and Health, Imperial College London, London, UK. [3]Centre for Atmospheric Science, Dept of Chemistry, University of Cambridge, Cambridge, UK. [4]Dept of Hygiene, Epidemiology and Medical Statistics, Medical School, National and Kapodistrian University of Athens, Athens, Greece. [5]National Heart and Lung Institute, Imperial College London, London, UK.

Correspondence: Benjamin Barratt, Environmental Research Group, Michael Uren Biomedical Engineering Hub, Imperial College London, White City Campus, London, W12 0BZ, UK. E-mail: b.barratt@imperial.ac.uk

@ERSpublications
Significant adverse associations were found between the respiratory health of COPD patients and their personal exposure to gaseous pollutants measured using portable sensors over 6 months. No significant associations were found for particulate pollutants. https://bit.ly/3aqMT6O

Cite this article as: Evangelopoulos D, Chatzidiakou L, Walton H, *et al*. Personal exposure to air pollution and respiratory health of COPD patients in London. *Eur Respir J* 2021; 0: 2003432 [https://doi.org/10.1183/13993003.03432-2020].

This single-page version can be shared freely online.

ABSTRACT Previous studies have investigated the effects of air pollution on chronic obstructive pulmonary disease (COPD) patients using either fixed-site measurements or a limited number of personal measurements, usually for one pollutant and a short time period. These limitations may introduce bias and distort the epidemiological associations as they do not account for all the potential sources or the temporal variability of pollution.

We used detailed information on individuals' exposure to various pollutants measured at fine spatiotemporal scale to obtain more reliable effect estimates. A panel of 115 patients was followed up for an average continuous period of 128 days carrying a personal monitor specifically designed for this project that measured temperature, nitrogen dioxide (NO_2), ozone (O_3), nitric oxide (NO), carbon monoxide (CO), and particulate matter with aerodynamic diameter <2.5 and <10 µm at 1-min time resolution. Each patient recorded daily information on respiratory symptoms and measured peak expiratory flow (PEF). A pulmonologist combined related data to define a binary variable denoting an "exacerbation". The exposure–response associations were assessed with mixed effects models.

We found that gaseous pollutants were associated with a deterioration in patients' health. We observed an increase of 16.4% (95% CI 8.6–24.6%), 9.4% (95% CI 5.4–13.6%) and 7.6% (95% CI 3.0–12.4%) in the odds of exacerbation for an interquartile range increase in NO_2, NO and CO, respectively. Similar results were obtained for cough and sputum. O_3 was found to have adverse associations with PEF and breathlessness. No association was observed between particulate matter and any outcome.

Our findings suggest that, when considering total personal exposure to air pollutants, mainly the gaseous pollutants affect COPD patients' health.

©The authors 2021. For reproduction rights and permissions contact permissions@ersnet.org
